# Inhibitory action on the production of advanced glycation end products (AGEs) and suppression of free radicals *in vitro* by a Sri Lankan polyherbal formulation Nawarathne Kalka

**DOI:** 10.1186/s12906-016-1178-x

**Published:** 2016-07-08

**Authors:** Chamira Dilanka Fernando, Diyathi Tharindhi Karunaratne, Sachith Dilshan Gunasinghe, M. C. Dilusha Cooray, Prabuddhi Kanchana, Chandani Udawatte, Pathirage Kamal Perera

**Affiliations:** College of Chemical Sciences, Institute of Chemistry Ceylon, Rajagiriya, Sri Lanka; Institute of Indigenous Medicine, University of Colombo, Rajagiriya, Sri Lanka

**Keywords:** Advanced glycation end products, Nawarathne Kalka, Free radicals

## Abstract

**Background:**

Advanced glycation end products (AGEs) and free radicals are inflammatory mediators and are implicated in many diseases such as diabetes, cancer, rheumatoid arthritis etc. Multi targeted poly herbal drug systems like Nawarathne Kalka (NK) are able to quench the overall effect of these mediators as they contain good combinations of phytochemicals that have least side effects in contrast to modern medicinal drugs. The objectives of this study were to evaluate phytochemical composition, free radical scavenging activity, cytotoxicity and the inhibitory action on the formation of AGEs by aqueous extract of NK.

**Methods:**

Total phenolic content (TPC) and total flavonoid content (TFC) were determined using Folin ciocalteu method and aluminium chloride assay respectively. Free radical scavenging activity was assessed by DPPH radical scavenging assay (DRSA), phosphomolybdenum reduction antioxidant assay (PRAA) and nitric oxide (NO) scavenging assay. Brine Shrimp Lethality (BSL) bioassay was performed as preliminary screening for cytotoxic activity. Inhibitory action on AGE formation was evaluated using fructose mediated glycation of bovine serum albumin using fluorescence spectroscopic method.

**Results:**

The TPC and TFC were 75.1 ± 3.0 mg/g gallic acid equivalents and 68.7 ± 7.8 mg/g epigallocatechin gallate equivalents. The DRSA yielded EC_50_ of 19.15 ± 2.24 μg mL^−1^ for NK. DRSA of NK extract was greater than butylated hydroxy toluene (EC_50_ = 96.50 ± 4.51 μg mL^−1^) but lesser than L-ascorbic acid (EC_50_ = 5.60 ± 0.51 μg mL^−1^). The total antioxidant capacity of NK as evidenced by PRAA was 106.4 ± 8.2 mg/g L-ascorbic acid equivalents. NK showed EC_50_ value of 99.3 ± 8.4 μg mL^−1^ in the NO scavenging assay compared to the standard ascorbic acid (EC_50_ = 7.3 ± 0.3 μg mL^−1^). The extract indicated moderate cytotoxic activity in the BSL bioassay. The extract showed effective inhibitory action on the formation of AGEs with EC_50_ values of 116 ± 19 μg mL^−1^, 125 ± 35 μg mL^−1^ and 84 ± 28 μg mL^−1^ in data obtained over three consecutive weeks respectively. Comparatively the reference standard, aminoguanidine at a concentration of 500 μg mL^−1^ demonstrated 65 % inhibition on the formation of AGE after one week of sample incubation.

**Conclusions:**

The results proved the potential of NK as a free radical scavenger, moderate cytotoxic agent and an inhibitor on the formation of advanced glycation end-products.

## Background

Traditional Sri Lankan System of Medicine (TSM) was established more than 3,000 years ago and it has been useful ever since for the treatment of various ailments [1]. In contrast to modern medicinal systems, polyherbal preparations have gained more attention for their multi-targeting ability via pathways that give fewer side effects [2]. These TSM drug systems consist of poly herbal formulations that can suppress painful symptoms associated with various ailments such as rheumatoid arthritis, diabetes and cancer [1].

Nawarathne Kalka (NK) is a similar poly herbal formulation which is used in TSM. This particular preparation contains components originating from 14 different plant species and it is mainly prescribed for gastrointestinal tract disorders such as diarrhea, abdominal pain, haematochezia, indigestion as well as for rheumatoid arthritis (RA) and other inflammatory conditions [3]. It consists of *Cedrus deodara* (Devadara), *Cuminum cyminum* (Suduru), *Eugenia caryophylla* (Karabu), *Ferula asafetida* (Perunkayam), *Glycyrrhiza glabra* (Walmi), *Myristica fragrans* (Sadikka), *Nigella sativa* (Kaluduru), *Picrorhiza kurroa* (Katukarosana), *Piper longum* (Thippili), *Trachyspermum roxburghianum* (Asamodagum), *Vernonia anthelmintica* (Sanninayam), *Zingiber officinale* (Inguru), *Terminalia bellirica* (Bulu), *Terminalia chebula* (Aralu), and bees honey. The ingredients and proportions of each component in NK and the parts of the plants used for its preparation are stated in Table 1 [3].

**Table 1:** Ingredients and proportions of Nawarathne Kalka [3]

Ingredients of Nawarathne Kalka	Part of the plant used	Proportions (weight basis)
1. *Cedrus deodara* (Vernacular name (VN): Devadara)	Bark	1
2. *Cuminum cyminum* (VN: Suduru)	Seeds	1
3. *Eugenia caryophylla* (VN: Karabu)	Flower bud	1
4. *Ferula asafetida* (VN: Perunkayam)	Resin	1
5. *Glycyrrhiza glabra* (VN: Valmi)	Stem	1
6. *Myristica fragrans* (VN: Sadikka)	Dried kernel of the seed	1
7. *Nigella sativa* (VN: Kaluduru)	Seeds	1
8. *Picrorhiza kurroa* (VN: Katukarosana)	Roots	1
9. *Piper longum* (VN: Thippili)	Dried fruit	1
10. *Trachyspermum roxburghianum* (VN:Asamodagum)	Seeds	1
11. *Vernonia anthelmintica* (VN: Sanninayam)	Seeds	1
12. *Zingiber officinale* (VN:Inguru)	Rhizome	1
13. *Terminalia bellirica* (VN: Bulu)	Fruit (outer cover)	13
14. *Terminalia chebula* (VN: Aralu)	Fruit (outer cover)	26
15. Honey	–	50

Diseases such as RA and diabetes are inflammatory mediated, and hence require anti-inflammatory medicines to suppress the overall effects associated with inflammation. Inflammation causes pro inflammatory cytokines to be elevated such as interleukine-17 (IL-17) and tumor necrosis factor alpha (TNF-α) [4], which would subsequently initiate the secretion of more inflammatory mediators such as cytokines like IL-6 and IL-8 [5] and colony stimulating factors like granulocyte macrophage colony stimulating factor (GM-CSF) [6]. This means that propagation of inflammation activates osteoclasts in RA-cartilages to initiate osteoclastogenesis which is common in pathophysiology of RA [7, 8]. The diabetes related complications such as retinopathy [9], nephropathy [10] are also driven by similar inflammatory pathways. Accumulation of advanced glycation end-products (AGEs) resulting from protein glycation are considered to be the initiators of these complications [10]. Advanced glycation end-products are formed due to the non-enzymatic reactions between sugars and proteins or nucleicacids [11, 12] and are associated with vascular related complications [13].

Oxidative stress is another factor that drives inflammation which can exert cytotoxic effects on tissues in the human body and hence there is a close association between oxidative stress and inflammation. Most common contributors of oxidative stress are hydroxyl radicals (^.^OH), nitric oxide (NO), superoxide anions (O_2_^.-^) and peroxynitrites (OONO^−^) and these contributors are known as reactive oxygen species (ROS) [14]. The ability to scavenge ROS is a useful quality that every anti-oxidant/anti-inflammatory drug must possess.

Suppression of the formation of ROS, AGEs and the secretion of cytokines altogether is the task of a multi targeted drug system rather than of a single targeted drug system. Hence the complex and complicated pathways by which the most dangerous diseases are associated with can be ameliorated by using the multi component formulations such as NK.

Due to lack of evidence on the pharmacologically important actions of the poly herbal formulation of NK towards suppression of various ailments, this study was focused towards investigation of NK for its phytochemical composition, antioxidant capacity and inhibitory action on formation of AGEs. Additionally, the cytotoxic effect of this herbal medicament was investigated.

## Methods

### Chemicals

2,2-diphenyl-1-picrylhydrazyl (DPPH), Glacial acetic acid, sulfanilamide, N-(1-napthyl)-ethylene diaminedihydrochloride (NEDD), sodium nitroprusside (SNP), L-ascorbic acid, potassium dihydrogen phosphate, disodium hydrogen phosphate, fructose, bovine serum albumin (BSA) and sodium azide were purchased from Sigma Aldrich USA.

### Preparation of aqueous extract of NK

NK was purchased from an Ayurvedic drug store. An amount of 15 g from 3 sachet packets of NK were pooled together and the contents were then dissolved in 400 mL of deionized water. This mixture was refluxed in dark for 3 h. The refluxed solution was filtered using Whatman no.1 filter paper and the filtrate was stored under 4 °C until further use. Prepared NK specimen (voucher number NK 102) was deposited in Department of Ayurveda Pharmacology and Pharmaceutics, Institute of Indigenous Medicine, University of Colombo, Rajagiriya, Sri Lanka.

### Determination of total phenolic content

The total phenolic content of the extracts were determined by Folin ciocalteu method [15]. The extract was diluted 50, 100 and 500 times using deionized water. Folin ciocalteu’s phenol reagent (1 N, 250 μL) was added to the sample (500 μL) and the mixture was allowed to stand at room temperature for 2 min. Sodium carbonate solution (10 %, 1.25 mL) was added and samples were incubated for 45 min in the dark at room temperature. The absorbances of the resulting solutions were measured at 760 nm against a blank prepared in same manner but replacing the extract with deionized water. The calibration curve was constructed using gallic acid standards (6 – 30 μg mL^−1^) and the total phenolic content of the extract was expressed as mg/g gallic acid equivalents (GAE).

### Determination of flavonoid content

The flavonoid content was measured by the aluminium chloride colorimetric assay [16]. The extract was diluted 3, 4 and 5 times using deionized water. The diluted extract (100 μL) was mixed with deionized water (400 μL) and sodium nitrite (5 %, 30 μL). After 5 min aluminium chloride (10 %, 30 μL) was added followed by sodium hydroxide (1 M, 200 μL) at 6^th^ minute. The total volume was adjusted to 1000 μL with deionized water and absorbance was measured at 510 nm against a blank prepared in similar manner but replacing the extract with deionized water. The calibration curve was plotted using (−)-epigallocatechingallate (EGCG) standards (300–1000 μg mL^−1^) and flavonoid content was expressed mg/g EGCG equivalents.

### 1,1-Diphenyl-2-picrylhydrazyl (DPPH) free radical scavenging activity

Free radical scavenging capacity of the NK extract was assessed by DPPH radical scavenging method according to a method published previously with slight modifications [17]. A concentration series was prepared by diluting the extract. DPPH reagent prepared in 96 % ethanol (100 μM, 2.0 mL) was added to diluted extract (0.5 mL) and the mixture was allowed to stand for 30 min in the dark. The scavenging activity by each concentration was quantified by measuring the decolourization of the resulting solutions at 517 nm. Deionized water was used as the blank. The control was prepared by mixing deionized water with DPPH. L-Ascorbic acid (1–20 μg mL^−1^) and butylated hydroxy toluene (BHT, 20 – 400 μg mL^−1^) were used as standard reference antioxidants. The results were expressed as percentage inhibition (%I) calculated according to equation 1 given below:

1 $$ \frac{\%\mathrm{I} = {\mathrm{A}}_{\mathrm{c}}\hbox{--}\ {\mathrm{A}}_{\mathrm{s}}\mathrm{x}\ 100\%}{{\mathrm{A}}_{\mathrm{c}}} $$

(where A_c_ = Absorbance of control and A_s_ = Absorbance of sample)

The effective concentration needed to scavenge 50 % of the DPPH free radical (Half maximal effective concentration, EC_50_) was calculated by regression analysis of the dose response curve plotted between percentage inhibition versus concentration of the test sample and the standard.

### Phosphomolybdenum reduction antioxidant assay

The total antioxidant capacity of the extract was evaluated based on the method developed by Prieto *et al.* [18]. The reduction of Mo (VI) to Mo (V) by the antioxidants present in the extract will subsequently form a green phosphate-Mo (V) complex at an acidic pH.

The extract was diluted 50, 100 and 500 times. Diluted extract (0.5 mL) was combined with 2.5 mL of reagent solution (0.6 M sulfuric Acid, 28 mM trisodium phosphate and 4 mM ammonium molybdate). The reaction mixture was then incubated at 95 °C for 90 min. Finally, after cooling the reaction mixture to room temperature, the absorbance was measured at 695 nm against a blank prepared in the same manner but using deionized water instead of extract. The calibration curve was constructed using L-ascorbic acid standards (25 – 100 μg mL^−1^) and the total antioxidant capacity of the above extracts was expressed as mg/g L-ascorbic acid equivalents.

### NO scavenging activity

The NO scavenging activity of NK extract was determined according to a method published previously [19]. Sodium nitroprusside (10 mM) solution was mixed with phosphate buffer (pH 7.4) in the ratio of 1:3 and kept for 20 min until the required aerobic conditions were obtained. Auto oxidation products (nitrites/nitrates) of NO generated by SNP were produced under these conditions. The SNP and buffer mixture (2.0 mL) was added to 1.0 mL of NK (0.1-19.8 mg mL^−1^) and the samples were incubated for 150 min at 25 °C.

Sulfanilamide (0.33 % in 20 % glacial acetic acid, 1.0 mL) was added to 0.5 mL of the previously incubated solution and allowed to stand for 5 min. Then 1.0 mL of NEDD (0.1 % w/v) was added to the mixture and further incubated for 30 min at 25 °C. The pink chromophore generated during diazotization of nitrite ions with sulphanilamide and NEDD was measured spectrophotometrically at 540 nm against a blank sample which consisted of NEDD, SNP and buffer only. The control was prepared by replacing NK with phosphate buffer which lacks a NO scavenger. L-Ascorbic acid was used as the positive control. Each analysis was performed in triplicates. The percentage inhibition (% I) of NO radicals by NK/positive control was calculated according to equation 1.

### Brine shrimp lethality bioassay

The cytotoxicity of the Nawarathne Kalka was determined using Brine Shrimp Lethality Bioassay [20]. Different concentrations of the extract (3.0 - 18.0 mg mL^−1^) were prepared by diluting the extract with deionized water. Diluted test sample (0.2 mL) was added to 24 well plates. Dimethyl sulfoxide (10 %, 0.05 mL) was added to each well and total volume was adjusted to 2.0 mL with artificial brine solution (25 g NaCl and 2.5 g MgSO_4_ dissolved in 1000 mL of deionized water, pH 8). The control solution was prepared by adding dimethyl sulfoxide and adjusting the total volume up to 2.0 mL with brine solution. Brine shrimp eggs were allowed to hatch in artificial brine solution for 24 h to obtain the nauplii. Ten living nauplii were transferred carefully to each well using a clean pasture pipette and kept for 18 h before monitoring the results. After 18 h, the number of living nauplii was counted. The experiment was conducted in triplicates. The percentage lethality (% L) was calculated according to equation 2 given below:

2 $$ \frac{\%\mathrm{L} = \left({\mathrm{N}}_{\mathrm{C}}\hbox{--}\ {\mathrm{N}}_{\mathrm{S}}\right)\mathrm{x}\ 100\%}{{\mathrm{N}}_{\mathrm{C}}} $$

(Where, N_c_ = Number of living nauplii in the control sample, N_s_ = Number of living nauplii in the test sample)

The effective concentration required to kill 50 % of the living nauplii with respect to the control (Half maximal lethal dosage, LD_50_) was calculated by the dose response curves plotted between %L versus concentration of the extract.

### Inhibitory action on the formation of Advanced Glycation End-products

The inhibitory action by NK on formation of AGEs was evaluated according to McPherson, 1988 with slight modifications [21]. Bovine serum albumin (BSA) solution (10mgmL^−1^) was prepared in phosphate buffered saline (pH 7.4) containing sodium azide (0.02 %) to minimize microbial activity during the experiment. Fructose (500 mM, 4.0 mL) was mixed with BSA solution (5.0 mL) and NK (0.1 – 19.8 mg/ml, 1.0 mL). Bovine serum albumin was used as the negative control. A sample containing BSA and fructose was used to induce the formation of advanced glycation end products. Samples containing only NK at respective concentrations were also run to measure any fluorescence emission caused by the endogenous substances present in NK. The fluorescence intensity of each mixture was measured at excitation and emission wavelengths at 355 nm and 460 nm respectively. Readings were obtained each week for a period of 3 successive weeks. Aminoguanidine was used as the reference standard. Each sample was analyzed in triplicates. The percentage inhibition (%I) caused by NK on the formation of AGEs was determined by equation 3 given below.

3 $$ \frac{\%\mathrm{I} = \left({\mathrm{F}}_{\mathrm{C}}\hbox{--}\ {\mathrm{F}}_{\mathrm{C}\mathrm{B}}\right)\ \hbox{--}\ \left({\mathrm{F}}_{\mathrm{S}}\hbox{--}\ {\mathrm{F}}_{\mathrm{S}\mathrm{B}}\right)\mathrm{x}\ 100\%}{\left({\mathrm{F}}_{\mathrm{C}}\hbox{--}\ {\mathrm{F}}_{\mathrm{C}\mathrm{B}}\right)} $$

(Where F_C_ = Fluorescence intensity of control with fructose, F_CB_ = Fluorescence intensity of blank of control without fructose, F_S_ = Fluorescence intensity of sample with fructose, F_SB_ = Fluorescence intensity of blank of sample without fructose)

### Statistical analysis

Results are presented as mean ± standard deviation (Mean ± SD) of at least three independent experiments. Statistical analysis including student’s *t*-test was performed using Microsoft Excel. Value of *p* < 0.05 was considered as significant.

## Results and Discussion

Phenolic compounds are considered to be the most important antioxidants and are widely distributed among various plant species. These phenols play important roles in plants such as protection against herbivores and pathogens, regulation of cell growth and cell division [22]. Polyphenols abound in natural products and in human diet and their roles in preventing chronic degenerative diseases have been proven in previous studies [23–25]. Nawarathne kalka being abundant with a collection of different plant species elicited a total phenolic content of 75.1 ± 3.0 mg/g GAE. This indicates the potential possessed by NK to prevent degenerative diseases including rheumatoid arthritis. Flavonoids are water soluble polyphenolic compounds which are extremely common and wide spread in the plant kingdom as their glycosides [22]. The documented biological effects of dietary flavonoids include anti-inflammatory, anti-allergic, antimicrobial, hepatoprotective, antiviral, antithrombotic, cardioprotective, capillary strengthening, antidiabetic, anticarcinogenic and antineoplastic effects [26]. The content of total flavonoid present in NK extract was 68.7 ± 7.8 mg/g epigallocatechin gallate equivalents which suggests that the flavonoids abundantly present in the extract may offer afore mentioned therapeutic benefits to humans. The results obtained for the determination of phytochemical composition of NK extract is given in Table 2.

**Table 2:** Phytochemical composition of NK aqueous extract

Experiment	Phytochemical Composition
Total Phenolic Content	75.1 ± 3.0 mg/g gallic acid equivalents
Total Flavonoid Content	68.7 ± 7.8 mg/g epigallocatechin gallate equivalents

**Fig. 1:**
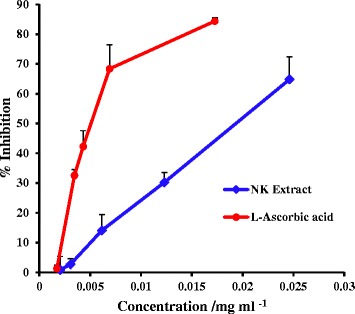
Dose response curves for NK extract and L-ascorbic acid for DPPH radical scavenging assay

1,1-Diphenyl-2-picrylhydrazyl (DPPH) free radical scavenging assay was used to determine hydrogen donating ability of the extracts. The EC_50_ values obtained from the dose response curves (Fig. 1) for the DPPH assay were 19.15 ± 2.24 μg mL^−1^, 5.60 ± 0.51 μg mL^−1^ and 96.50 ± 4.51 μg mL^−1^ for NK extract, L-ascorbic acid and BHT respectively. This indicates that the antioxidant potential of the NK extract was higher than BHT but lesser than L-ascorbic acid.

Phosphomolybdenum reduction antioxidant assay is a single electron transfer system which is useful in measuring the capacity of an antioxidant in reduction of an oxidant which changes its color when reduced. A higher degree of color formation indicates higher reducing power of the antioxidant [27]. NK extract demonstrated an antioxidant capacity of 106.4 ± 8.2 mg/g L-ascorbic acid equivalents in this assay. This proves that the extract has a higher reducing power to almost neutralize many oxidants generated *in vivo* as well as arising from exogenous sources.

**Fig. 2:**
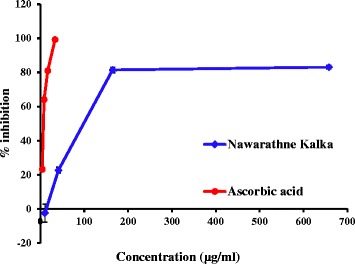
Dose response curve for percentage inhibition of NO production by L- ascorbic acid and NK

Nitric oxide is mainly produced by many isoforms of nitric oxide synthases (NOS) that are mainly inducible-NOS, endothelial-NOS and neuronal-NOS [28]. Nitric oxide synthases release NO under different stimuli for different purposes [28]-[29]. The over production of NO is similarly dangerous as impaired production of NO [30–33]. In this study only the suppression of over production of NO by NK is considered. Nitric oxide scavenging ability for both the positive control (L-ascorbic acid) and the NK water extract were considered and the percentage inhibitions calculated for the respective concentrations were plotted against each other and are shown in Fig. 2. Water extract of NK showed higher EC_50_ of 99.3 ± 8.4 μg mL^−1^ than the respective positive control which was L- ascorbic acid at EC_50_ of 7.3 ± 0.3 μg mL^−1^. This suggests that compared to the positive control, the water extract of NK showed moderate NO scavenging activity. This activity may be helpful for NK to act neutral under lower NO production conditions while acting as a moderate NO scavenger under over production of NO. However, this activity should be further analyzed and researched.

**Fig. 3:**
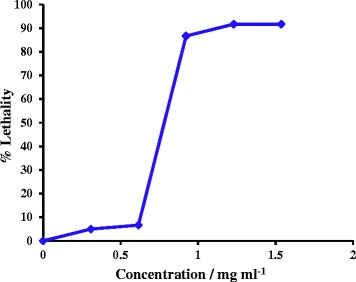
Cytotoxic effect of NK extract on shrimp nauplii

The brine shrimp lethality bioassay represents a rapid, inexpensive and simple bioassay for testing plant extracts’ bioactivity which in most cases correlates reasonably well with cytotoxic and anti-tumor properties [34]. This assay has been widely used for the isolation of bioactive compounds from plant extracts and for estimating the extent of *in-vivo* lethality on *Artemia salina* (brine shrimp) [34, 35]. Drugs derived from plants have many anticancer properties like topoisomerase-I and topoisomerase- II inhibition, thymidylate synthase inhibition, apoptotic effect, interaction with cyclin dependent kinases, anti-mitotic properties etc., leading to lethality with exposure time and dosage of the drug [36]. The aqueous extract of NK indicated LD_50_ value of 807.6 ± 221.0 μg mL^−1^as determined according to the dose response curve depicted in Fig. 3. The extract demonstrated percentage lethality greater than 85 % at concentrations above 900 μg mL^−1^. This indicates that the aqueous extract of NK possess moderate cytotoxic activity. However toxicity studies with mammalian cancer cell line versus normal cell line remains to be established in order for better understanding of the cytotoxicity that NK may selectively cause towards cancerous cells compared to normal cells.

**Fig. 4:**
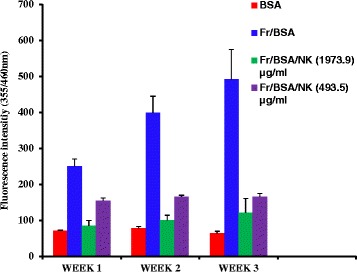
Fluorescence intensity versus time of sample measurements. Respective blanks solution with BSA only, showed fluorescence intensities at constant levels throughout the three weeks (red bars). Whereas when the blanks samples were introduced with fructose (Fr) the formation of fluorescence AGE got induced hence the fluorescence intensity increased significantly (*p* < 0.05) (blue bar). But when samples were simultaneously treated with NK a significant decrease (*p* < 0.05) in the fluorescence intensities was indicated in a concentration dependent manner (green & purple bars) compared to the sample treated with BSA and fructose

**Fig. 5:**
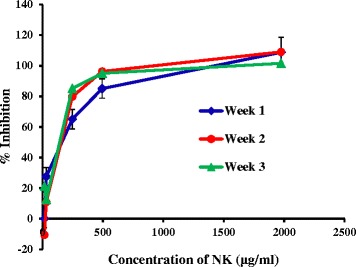
Overlay of the dose response curves for percentage inhibition on advanced glycation end-product formation by the aqueous extract of NK (Week1 – Week3)

Formation of AGEs is induced under conditions such as hyperglycemia, dyslipidemia and oxidative stress. This phenomenon can lead to complications related to diabetes such as retinopathy and nephropathy. Disturbance in the formation of such AGEs can contribute in suppressing any complications related to AGEs [37]. Fluorescence emission caused by AGEs and emission from AGEs under treatment conditions with NK at three concentration levels over a period of three weeks are shown in Fig. 4. There was a significant increase in fluorescence intensities (*p* < 0.05) observed for the BSA samples treated along with fructose compared to the BSA (only) samples (Fig. 4). This indicates the formation of fluorescent AGEs occurred over the time period due to the glycation of BSA with fructose [38]. However, when the samples of BSA along with fructose were treated separately with 493.5 μg mL^−1^ and 1973.9 μg mL^−1^ concentrations of the NK extract, it was observed that the fluorescence emission resulting due to AGEs are significantly reduced (*p* < 0.05) with respect to the samples treated with BSA and fructose (Fig. 4). This in turn suggests the disruption caused on AGE formation by the components present in the aqueous extract of NK. NK in this study, not only suppressed the formation of fluorescent AGEs, but also continued suppressing such AGE from forming throughout the three weeks, as evidenced by the dose response curves obtained during this time period (Fig. 5). The EC_50_ values obtained for the dose response curves corresponding to Week 1, Week 2 and Week 3 were 116 ± 19 μg mL^−1^, 125 ± 35 μg mL^−1^ and 84 ± 28 μg mL^−1^ respectively. The reference standard, aminoguanidine at a concentration of 500 μg mL^−1^ demonstrated 65 % inhibition on the formation of AGE after one week of sample incubation. This provides enough support to consider NK as a possible anti glycation drug even though the precise mechanism of how NK suppress the formation of AGE is not known. Advanced glycation end-product formation is due to the non-enzymatic reaction between sugars and proteins. This reaction forms imines/Schiff’s bases. These Schiff’s bases will subsequently enter an irreversible rearrangement called Amadori rearrangement, and will become Amadori products eventually [39]. Considering the reversibility of the two steps of AGE formation, NK might be possessing the ability to terminate the formation of irreversible Amadori products, hence capable of remaining as an anti glycation drug for a long period.

Several studies conducted with different types of honey have proven their antioxidant properties. This is due to the compounds present such as vitamin C, monophenolics, flavonoids and polyphenolics. Antioxidant compounds like caffeic acid, chrysin, galangin, quercetin, acacetin, kaempferol, pinocembrin, pinobanksin, apigenin and enzymes like glucose oxidase and catalase are found to predominate in most of the types of honeys [40]. They have received special attention due to their role in preventing diseases associated with oxidative stress such as cancer, cardiovascular diseases, inflammatory diseases and infections [40, 41]. Honey being a main ingredient in the formulation of NK may be responsible for this therapeutic potential of the formulation itself and causing a synergistic effect along with phyto-constituents derived from plant materials to enhance the activity of the medicament. The next most abundant ingredients *Terminalia belerica* and *Terminalia chebula* present in NK have been scientifically proven for many biological activites including antioxidant and anti-diabetic effects [42, 43]. NK being a polyherbal formulation comprising of these two ingredients also would have added to and enhanced the overall effects of NK. Future studies will be focused at identification and quantification of individual compounds present in NK.

## Conclusion

Our findings provide evidence of potent antioxidant activity, moderate NO scavenging activity and cytotoxic effects as well as the ability to inhibit the formation of advanced glycation end products possessed by the poly herbal formulation Nawarathne Kalka. This can be attributed to very high levels of phenolic and flavonoid compounds being present, thus justifying the use of this particular herbal remedy in the treatment of various inflammatory conditions including arthritis in the Traditional Sri Lankan System of Medicine. However further studies including identifying potent individual chemical components present in NK, their mechanistic pathways of action and clinical trials should be conducted to understand the holistic effects caused by this poly herbal medicament on human body.

## Abbreviations

% I, Percentage inhibition; ^.^OH, Hydroxyl radicals; AGEs, Advanced glycation end products; BHT, Butylated hydroxy toluene; BSA, Bovine serum albumin; BSL, Brine Shrimp Lethality bioassay; DPPH, 2,2-diphenyl-1-picrylhydrazyl; DRSA, DPPH radical scavenging assay; EC_50_, Half maximal effective concentration; EGCG, (−)-epigallocatechingallate; GAE, Gallic acid equivalents; GM-CSF, Granulocyte macrophage colony stimulating factor; IL-17, Interleukine-17; LD_50_, Half maximal lethal dosage; NEDD, N-(1-napthyl)-ethylene diaminedihydrochloride; NK, Nawarathne kalka; NO, Nitric oxide; O_2_^.-^, Superoxide anions; OONO^−^, Peroxynitrites; PRAA, Phosphomolybdenum reduction antioxidant assay; RA, Rheumatoid arthritis; ROS, Reactive oxygen species; SNP, Sodium nitroprusside; TFC, Total flavonoid content; TNF-α, Tumor necrosis factor alpha; TPC, Total phenolic content; TSM, Traditional Sri Lankan System of Medicine
